# Ethics takes time, but not that long

**DOI:** 10.1186/1472-6939-8-6

**Published:** 2007-05-24

**Authors:** Mats G Hansson, Ulrik Kihlbom, Torsten Tuvemo, Leif A Olsen, Alina Rodriguez

**Affiliations:** 1 Centre for Bioethics at Karolinska Institutet and Uppsala University, Department of Public Health and Caring Sciences, Uppsala University, Uppsala, Sweden; 2 Department of Humanities, Örebro University, Örebro, Sweden; 3 Department of Women's and Children's Health, University Children's Hospital, Uppsala, Sweden; 4 Department of Pediatric Surgery, University Children's Hospital, Uppsala, Sweden; 5 Department of Public Health Science and General Practice, Oulu University, Oulu, Finland; 6 Department of Psychology, Uppsala University, Uppsala, Sweden

## Abstract

**Background:**

Time and communication are important aspects of the medical consultation. Physician behavior in real-life pediatric consultations in relation to ethical practice, such as informed consent (provision of information, understanding), respect for integrity and patient autonomy (decision-making), has not been subjected to thorough empirical investigation. Such investigations are important tools in developing sound ethical praxis.

**Methods:**

21 consultations for inguinal hernia were video recorded and observers independently assessed global impressions of provision of information, understanding, respect for integrity, and participation in decision making. The consultations were analyzed for the occurrence of specific physician verbal and nonverbal behaviors and length of time in minutes.

**Results:**

All of the consultations took less than 20 minutes, the majority consisting of 10 minutes or less. Despite this narrow time frame, we found strong and consistent association between increasing time and higher ratings on all components of ethical practice: information, (β = .43), understanding (β = .52), respect for integrity (β = .60), and decision making (β = .43). Positive nonverbal behaviors by physicians during the consultation were associated particularly with respect for integrity (β =.36). Positive behaviors by physicians during the physical examination were related to respect for children's integrity.

**Conclusion:**

Time was of essence for the ethical encounter. Further, verbal and nonverbal positive behaviors by the physicians also contributed to higher ratings of ethical aspects. These results can help to improve quality of ethical practice in pediatric settings and are of relevance for teaching and policy makers.

## Background

Doctor – patient communication during the medical consultation is central for effective care; however, it has been known for some time that a sizable number of patients in hospital (41%) and general practice (28%) settings are dissatisfied with communication [1]. Dissatisfaction often stems from inadequate explanations and feelings of being devalued or rushed [2]. Patient understanding and respect for integrity are not just elements of good communication, but also are the cornerstone of ethical exchange.

The requirement to obtain informed consent is universal within medicine today, even though information and consent procedures vary [3-7]. It is further clear that most patients want to be informed and that many also want to participate in medical decision-making [8-10]. Accordingly, there is a strong emphasis on patients' understanding of procedures, risks, benefits and alternative therapies whether participating in medical treatment or in clinical trials [11-13].

The same ethical focal points are emphasized when children are treated or are research participants [14-17]. It is essential that the consultation be allocated enough time so that both parents and children can have an opportunity to ask questions [18], thus augmenting the exchange of information, understanding, and issues of voluntariness and consent. From this perspective, child age is crucial, because children's capability to assent depends on their level of maturation and development [19]. The age limit for soliciting assent in addition to parental consent is set at 7 years (e.g. according to Swedish Regional Ethics Boards), however one study found that children younger than 9 years understood poorly or not at all [20]. In contrast, young children and even infants have an understanding of basic emotions conveyed by others' expressions and speech quality, i.e. nonverbal behavior [21].

The current study sought to explore elements of communication in pediatrics between doctors and patients/parents in relation to ethical practice that can guide future clinical training. According to a recent review [22], studies examining physician verbal and nonverbal behaviors during actual consultations are few and there is a great need to further empirically study such behaviors particularly for educational purposes [23]. Because our interest was in finding "good examples" we focused only on positive behaviors. This study is the first to record physician nonverbal behaviors in relation to ethical practice.

We observed behavior during a routine consultation for inguinal hernia in a pediatric surgery outpatient clinic. We selected this procedure because it is routine in that it is easy to treat and relatively common. However, successful treatment requires surgery which necessitates the need for clear information and decision-making, thus highlighting ethical practice. Further, suspected inguinal hernia entails a brief physical examination of the child's intimate areas that requires particular sensitivity on the part of the physician to insure respect for children's integrity. The term respect for integrity is vague and is often used in different ways. Here, we follow one main understanding that reflects respect to the patient by the physician in terms of sensitivity to patients' mental sphere (e.g. beliefs, desires, and decisions) and corporal sphere (e.g. unwanted physical contact). We were also interested in the amount of time spent on the consultation. A recent study shows that time spent in the consultation is not a top priority [24], however, we hypothesized that time would be important in pediatric cases. Time may be thought to be important for the ethical quality of a consultation based on the fact that giving information as well as ensuring patient understanding are time consuming processes. It may also take time to create an atmosphere in which patients feel secure, particularly children who may be unfamiliar with the situation. Thus, our aims were to measure the occurrence of positive behaviors by physicians and the length of time spent on the consultation and to determine if these were related to ethical practice as appraised by observers.

## Methods

The study took place at the pediatric surgery outpatient clinic at a university hospital serving a population of 300,000 local residents and a region of 1.3 million for referred cases. Patients had been referred to the clinic by general practitioners, school health services or via emergency room for assessment of possible inguinal hernia. Patients were consecutively recruited at the time of their scheduled appointments. The attending nurse introduced the study and obtained consent from parents and assent from older children. All consultations were recorded on digital video disc (DVD).

Surgeons had been informed about the purpose of the study prior to its initiation. However, no details were provided regarding the behaviors that would be observed. All participants, patients and surgeons alike, were guaranteed confidentiality and that no one outside the research team would view the video discs. Because the study took place at a teaching hospital, medical students were present in some of the consultations. The study was approved by the regional ethics committee at Uppsala University Hospital.

### Measures

We observed specific physician nonverbal behaviors directed towards the parent and positive behaviors occurring specifically during the physical examination directed towards the child. Behaviors selected for observation were chosen because they are considered to exemplify good doctor – patient communication reflecting empathy and listening skills [e.g. [25-27]]. Physician-initiated nonverbal behaviors directed towards the parent were recorded. We selected to concentrate on positive behaviors that reflect attentiveness or interest and consisted of looking at the parents during the consultation, paying full attention to parents while listening (e.g. not reading/writing in the medical record), waiting for the parents' attention before speaking, relaxed posture, having opened or approachable posture, speaking clearly, smiling or chuckling, physical contact with the parent during greeting or departure e.g. shaking hands, and being at eye level with the parent.

Positive behaviors occurring specifically during the physical examination directed towards the child included: interacting with the child before initiating the physical examination, approaching the child, being at eye level, making an effort to put the child at ease, showing responsiveness to child's mood, effectively dealing with child's mood, distracting, speaking softly, touching child softly, not being intrusive, inviting parents to stay close by, asking for permission to examine, informing what is/will be done during the examination, monitoring the child's state by asking how it is going, providing praise, and signaling that the child may re-dress.

Each behavioral occurrence was tallied and summed across both observers. After independent observations were made, observers watched the tapes together and agreed on the number and type of behaviors that were recorded.

We related behaviors to elements of ethical practice, i.e. the outcome variables. We defined ethical practice as consisting of informed consent (provision of information, understanding), respect for integrity (sensitivity, responsiveness, and respectfulness) and patient autonomy (decision-making). Global ratings of the aspects of ethical practice were made by the research team using five point scales. The global ratings were not meant to correspond to particular behaviors, but instead were general appraisals based on the entire consultation. Provision of information was rated as *lacking*, *insufficient*, *only a few key points provided, several key points provided*, and *thorough*. Parents' apparent understanding of the information provided by the surgeon was coded as *lacking*, *very little*, *partial understanding*, *mostly*, and *completely*. Respect for integrity was rated using three questions concerning general politeness, actively showing efforts to set the stage for a respectful encounter, and showing sensitivity in responding to parents' needs in a respectful manner. These three questions were also rated on a five-point scale (ranging from lacking to very much) and were pooled for analysis by taking the mean of the three questions per observer. Finally, we coded to what extent the surgeons actively involved parents in the decision making process. The ratings of the ethical aspects were averaged across raters for each consultation.

Observers rated child mood during the examination using a three point scale, (1 = calm, 2 = somewhat upset, 3 = very upset). The duration of the consultation was recorded in minutes.

Prior to the consultation parents were asked to provide demographic characteristics. Parents rated the perceived risk to their child's health of having an inguinal hernia as well as their own level of anxiety.

### Analyses

The consultations were coded in two steps. The first viewing was done independently by three of the authors, two ethicists (M.G.H., U.K.) and one psychologist (A.R.) who made global assessments of the ethical components. The second viewing of the consultations was done at least two months later when specific behaviors were tallied (U.K. and A.R.) and summed across raters.

We produced statistical analyses with SAS version 8.2 (SAS, Cary, NC, USA); all statistical tests of hypotheses were two sided at p < .05. The predictor variables were the total number of physician behaviors and time. The outcome variables were the global assessments of the ethical components (provision of information, understanding, respect for integrity and decision-making). Spearman correlation analyses were used to check inter-rater reliability of the global assessments: provision of information, understanding, respect for integrity, and decision making concerning the parents, and respect for children's integrity. The strength of the association is appropriately measured using Spearman correlations when data are solely based on an ordinal scale, such as the global assessments. We measured the degree of inter-rater agreement by examining the strength of Spearman correlations among the raters. Pearson correlation coefficients were used to assess the associations between coded nonverbal behaviors toward parents at any time during the consultation, consultation time, and the global assessments. Further we assessed whether the associations were modified by other variables pertaining to the consultation (e.g. physician gender). For children, we focused on the association between physician behaviors during the physical examination and global ratings of respect for child integrity. To investigate the amount of independent variance explained by physician behaviors and consultation time on the aspects of ethical practice we used multiple regression models.

## Results

**Figure 1:**
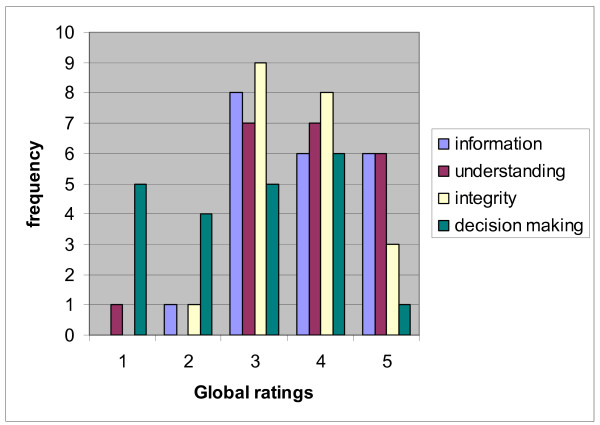
Sample distribution of ethical component ratings.

The study sample included 21 patients and their parents out of 29 who were eligible (unwillingness to be filmed was the overriding reason for nonparticipation) and eight surgeons (five men and three women). All surgeons who assessed inguinal hernia cases during the study period participated. Table 1 shows study participants' background and characteristics of the consultation. The average age of children in the study was under four years (M = 3.8, SD = 3.2). The global ratings between coders were significantly correlated and indicated acceptable inter-rater agreement. Spearman correlation coefficients ranged between .61 to .71 for information, .50 to .79 for understanding, .44 to .76 for respect for integrity, and .86 to .88 for degree of participation in decision making among the three raters. These coefficients indicate that agreement was moderate to strong. Further, we noted that raters were no more than one point in disagreement with each other, which indicates acceptable agreement. Figure 1 shows the frequency distributions for each of the ethical components based on the means of the three raters. The shape of the distributions for information, understanding, and respect for integrity were somewhat positively skewed. The distribution for involving parents in decision making showed that many parents did not participate actively.

**Table 1:** Characteristics of study participants

Background Characteristics	Frequency	Mean
Patient Age		3.2
		Range = 0–12
Patient		
Male	18	
Female	3	
Accompanying parent's age		39
		Range = 24–57
Parent occupation		
Blue collar	11	
White collar	4	
Unemployed/student/retired	3	
Parental perceived risk*		.7
Parental worry*		1.5
Characteristics of Consultation		
Medical students present	6	
Uncertainty of diagnosis	6	
Decision to operate	14	

*rated on a five point scale where 0 = not at all and 4 = very much

The occurrence of nonverbal behaviors varied widely between consultations and ranged from 3 to 66 (M = 28, SD = 19). The most frequently occurring behavior was speaking clearly.

Time varied across consultations from as little as one minute to as much as 6 minutes before the physical examination. Examination times ranged between 1 to 4 minutes. Over 52% of the cases were examined in 1 minute or less and only one case took 4 minutes. Post-examination time ranged between 1 to 10 minutes with more than 85% of the cases taking 5 minutes or less. The total time for consultations ranged between 3 to 19 minutes (M = 8.2, SD = 3.4). More than 85% of the consultations lasted 10 minutes or less.

Correlation analyses showed that the occurrence of physician nonverbal behaviors directed towards the parents was related to all ethical components we measured, as seen in Table 2. The length of time spent on the consultation was also positively related to ratings of ethical aspects; however, the time spent post-physical examination was clearly most important.

**Table 2:** Pearson correlation analyses of ethical components directed towards the parent in relation to consultation characteristics

	Information	Understanding	Respect for Integrity	Decision Making
Positive nonverbal behaviors by physicians	.54**	.46*	.60**	.49*
Time:				
pre-examination	.37	.39	.45*	.41
examination	.26	.40	.36	.60**
post-examination	.81***	.66**	.77***	.71***
total	.66**	.67**	.75***	.77***
Medical student present	-.07	-.25	-.23	-.28
Uncertainty of diagnosis	.43*	.34	.40	.62**
Child age	.22	.24	.31	.34
Physician gender	.20	.47*	.42	.44*

*p < .05

**p < .01

***p < .001

Total time of the consultation was related to the positive nonverbal behaviors directed towards parents, r = 40, p < .07, although the association did not quite reach significance possibly due to limited sample size, it suggests that as time increased so did the amount of positive behaviors. Time spent prior to the examination was clearly related to the amount of positive nonverbal behaviors, r = .57, p < .001. Time spent during the examination was completely unrelated to the amount of positive behaviors occurring specifically during the physical examination directed towards the child (r = -.02).

We explored whether physicians were "on their best behavior" when medical students were present and found no significant correlations. Child age was not related to the ethical components, but the trend suggests that as age increased ethical ratings were more positive. Consultations in which physicians were women had significantly higher ratings on understanding and decision making than when physicians were men.

In order to discern the amount of independent variance explained by physician initiated nonverbal behaviors and consultation time on the ethical components, we ran a series of multiple regression analyses controlling for potential confounders, i.e. variables that were found to be significantly correlated with the outcome (see Table 2). As seen in Table 3, total consultation time was significantly associated with higher ratings on all aspects of ethical practice we investigated and accounted for a substantial amount of independent variance. Positive nonverbal behaviors initiated by physicians were associated with higher ratings of respect for integrity. Nonverbal behaviors seemed to be marginally related to the other ethical components; however, it may be that the small sample size precluded significance from being reached. None of the confounders (from Table 2) were significant with the exception of uncertainty of diagnosis in relation to decision making. Physicians were more inclined to set the stage for joint decision making when they were uncertain if the child had hernia or not.

**Table 3:** Multiple regression analyses

	Positive nonverbal behaviors by physicians			Total consultation time				controlled for*
	t	p<	Beta	t	p<	Beta	Model Adjusted R^2^	

Information	1.96	.07	.35	2.12	.05	.43	.48	Uncertainty of diagnosis
Understanding	1.03	ns	.19	2.26	.01	.52	.44	Physician gender
Respect for Integrity	2.40	.03	.36	4.03	.001	.60	.63	--
Decision Making	1.58	ns	.23	2.50	.02	.43	.68	Physician gender, Uncertainty of diagnosis**

*controlling for factors that were significant in Table 2

** Uncertainty of diagnosis significant (t = 2.64, p < .02, β =.39)

The physical examination was visibly stressful for many children. Observers rated 53% of the children as calm, 29% somewhat upset, and 18% as very upset. We analyzed whether positive behaviors by physicians were important for respect for children's integrity using Pearson correlation analyses. We found a strong correlation between positive behaviors during the examination and respect for children's integrity, r = .70, p < .001. Child's mood during the examination seemed to be somewhat related, but statistical significance was not reached (r = -.36, ns). On the other hand, child age was moderately related to ratings of respect for integrity (r = .57, p < .01). Respect for children's integrity was not related to the time spent on the examination (r = .05, ns), but to the total time of the consultation (r = .60, p < .001). Physician gender and presence of a medical student were both unrelated. We used multiple regression analysis to examine respect for children's integrity using the variables we found significant in the correlation analyses. Positive behaviors (t = 3.63, p < .001, β =.49), total consultation time (t = 2.63, p < .01, β =.35) and child age (t = 2.27, p < .03, β =.31) significantly contributed to respect for children's integrity ratings and together explained 68% of the variance.

## Discussion and conclusion

The current study provides preliminary information on the importance of time in the consultation and positive behaviors by physicians that can guide future research and is immediately beneficial to clinicians, researchers, and health-care policy makers. All of the consultations we studied took less than 20 minutes, the majority consisting of 10 minutes or less. Despite this narrow time frame, we found strong and consistent association between increasing time and higher ratings on all components of ethical practice we studied, especially respect for integrity.

Examination and diagnosis of inguinal hernia is usually quite brief for an experienced surgeon. The positive signs are easy to detect either physically or through the patient history. Therefore, the amount of time spent on consultations in this study is not unusual and compatible with the time allocated for appointments at the study site. The patients in this study were all healthy children and did not demand further work-up for other complications.

There is disagreement amongst ethicists about the necessity for empirical investigations and their impact on normative medical ethics [28,29]. Our view is that empirical investigations such as this one are necessary to identify the non-ethical factors that underlie sound ethical praxis in clinical settings. Empirical studies are important because they are part of the process in which open-ended concepts can be tested, revised, or challenged. Previously, doctors' behaviors related to friendliness, courtesy, and the like have been shown to be related to patient satisfaction [30]. We offer evidence that doctors' behavior, including nonverbal behavior, is important from an ethical perspective and that these behavioral subtleties are instrumental in setting the stage for a respectful encounter.

Physical examination was stressful for the children. Our study indicates that a positive response to these situations will draw not so much on verbal as on nonverbal behavior that put the child at ease. Physicians who could serve as role models expressed relaxed concern and respect for the child and his/her parent. The video-recorded observations captured aspects of communication that relate to ethical practice, which would otherwise be difficult to assess using pencil-and-paper assessments. However, our study is limited by the small sample size that did not permit the analysis of individual behaviors. Our aim was not to offer a recipe of specific behaviors. Such would stifle the doctors' individuality and responsiveness to the particular situation at hand. The nonverbal behaviors we studied in the consultation all reflect genuine attentiveness and interest. Specific behaviors may very well vary, but the message is clear that the physician should not "act" interested, but convey interest via posture and in nonverbal quality of speech. Some of these behaviors may be culturally sensitive so that interest and openness is conveyed or perceived differently according to cultural background.

Our findings show robust relations between time and positive nonverbal behaviors in relation to ethical practice, therefore, it is unlikely that our results were due to chance. On the other hand, statistical power to detect true relations among the variables was limited due to our small sample size. Lack of statistical significance points to uncertainty, even though relations were in the expected direction, replication in future studies is required.

The generalizability of our study is somewhat limited because we examined only one type of medical case. However, we made this choice from a statistical standpoint to limit variability associated with examining a variety of medical cases. We also made the choice to focus on inguinal hernia because it is particularly related to issues of respect for integrity and because it is surgically treated; issues on informed consent are highlighted.

Despite the limitations, this study provides support for the importance of physician behaviors and the time spent on the consultation for the ethical exchange, which have not previously received much research attention. The insights provided by the present research should serve as an impetus for more detailed analysis of physician behaviors. Physicians and policymakers strive for efficiency in healthcare, which sometimes can translate into less time being allocated to the medical consultation. Consequently, some doctors may feel rushed or pressured and perhaps cut off the consultation prematurely. This may show in their nonverbal behavior. Time in itself, however, may not be key, but rather the behaviors that fill the time. More time gives the opportunity for more positive behaviors, as we found. Further studies should investigate whether time may be related to a greater opportunity for parents and patients to ask questions or perhaps just for pauses in which an opportunity to think about the message is provided. Our results show that just a little bit of time and physicians imparting relaxed interest make a big difference for ethical practice. The price for a few minutes of physician time may be far lower than the price of ethical transgressions.

## Competing interests

There are no affiliations or financial involvement with any organization or entity with a direct financial interest in the subject matter or materials discussed in the manuscript.

Authors have no financial or non-financial competing interests to disclose.

## Authors' contributions

MGH and AR conceptualized the study and TT and LO provided important insights. AR designed, planned and supervised the data collection and carried out the analyses. MGH and UK contributed to the construction of the observational coding system, data collection and assisted in interpretation of results. MGH acquired funding. MGH and AR drafted the manuscript and AR had overall responsibility. All authors contributed to the critical revision and approved the final version for publication.

## Pre-publication history

The pre-publication history for this paper can be accessed here:


